# *Qingryllus jiguanshanensis* sp. n. from Sichuan, China, the second species of *Qingryllus* (Orthoptera, Gryllidae)

**DOI:** 10.3897/zookeys.663.11494

**Published:** 2017-03-27

**Authors:** Haoyu Liu, Dongxiao Zhang, Fuming Shi

**Affiliations:** 1 The Key Laboratory of Zoological Systematics and Application, College of Life Sciences, Hebei University, Baoding 071002, Hebei Province, China

**Keywords:** Gryllinae, *Qingryllus*, taxonomy

## Abstract

A second species of *Qingryllus* Chen & Zheng is described and illustrated, *Q. jiguanshanensis* **sp. n.**, from Sichuan, China. This new species is similar to *Q. striofemorus* Chen & Zheng, 1995, but differs from the latter by the posterior margin of pronotum being distinctly widened, the veins of tegmina yellowish-white only on lateral side of dorsal area, and the epiphallus distinctly widened posteriorly. A distribution map of the species of this genus and habitus photographs of the new species are presented.

## Introduction

The genus *Qingryllus* was established by Chen and Zheng (1995) for *Q. striofemorus* Chen & Zheng, 1995, based on female specimens. Li and Zheng (2001) described the male characters of *Q. striofemorus*, with limited distribution in Ningshan (China: Shaanxi), this genus remains monotypic until the present time (Eades et al. 2016).

**Figure 1.**
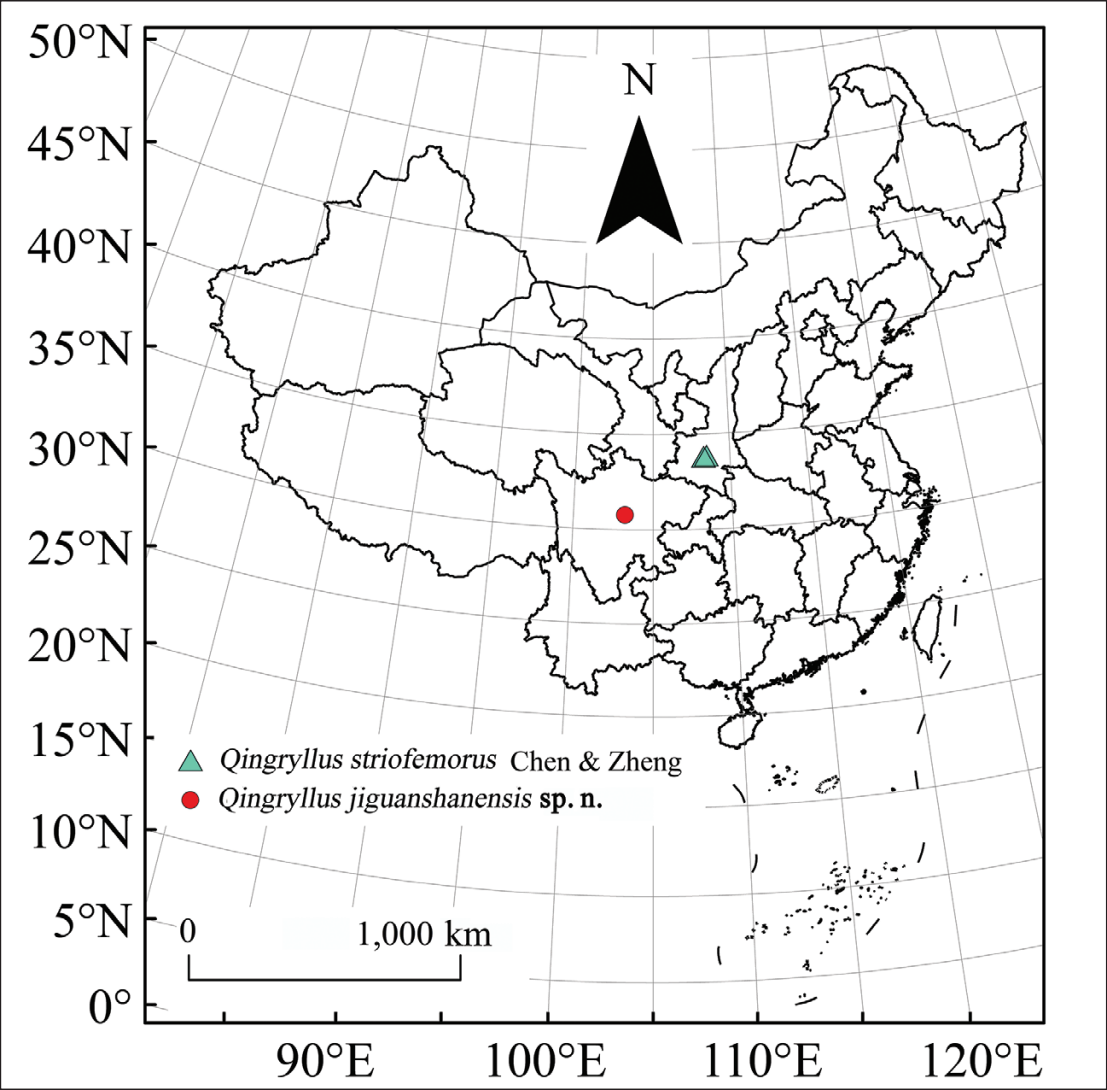
Distribution map of the genus *Qingryllus* Chen & Zheng.

In our recent study on Gryllinae from Sichuan, China, a new species of *Qingryllus* was discovered and it is described here under the name of *Q. jiguanshanensis* sp. n. There are two species of *Qingryllus*, both occurring in China (Figure 1).

The genus *Qingryllus* differs from most groups of Gryllinae by the fore tibia without tympanum and tegminal venation similar in both sexes. In Gryllinae, the genus *Qingryllus* has the greatest similarity to *Goniogryllus* Chopard, 1936, but differs from the latter by the tegmina, developed hind wings, and hind tibiae with four pairs of dorsal spines on both sides. *Goniogryllus* is generally without wings or with very short tegmina, and the hind tibiae usually has three dorsal spines (Chen and Zheng 1995). Meanwhile, the two genera have different microhabitat preferences, with *Qingryllus* living in trees and shrubs and *Goniogryllus* living in the litter layer.

## Materials and methods

Specimens examined were collected using sweeping method in trees and shrubs. The type specimens of the new species have been deposited in the Museum, Hebei University, Baoding, China (MHBU).

The male genitalia were dissected and cleared in 10% KOH solution. All morphological structures were photographed using a Leica M205A microscope. Images of multiple layers were stacked using Combine ZM. The distribution map was constructed using the software package ArcGIS 10.2 (ESRI, Redlands, CA, USA), based on localities of the specimens examined for this study and those mentioned in literature (Chen and Zheng 1995; Li and Zheng 2001).

## Taxonomy

### Qingryllus jiguanshanensis sp. n.

Taxon classificationAnimaliaOrthopteraGryllidae

http://zoobank.org/6F867825-77BF-4133-97E2-4B331CF3A1DE

Figures 2 , 3 , 4

#### Type material.

Holotype ♂: CHINA: Sichuan, Chongzhou, Jiguanshan, Shaoyaogou, 29.V.2016, leg. Fuming Shi. Paratypes: 1♂, 1♀: same data as the holotype.

#### Description.

**Figure 2.**
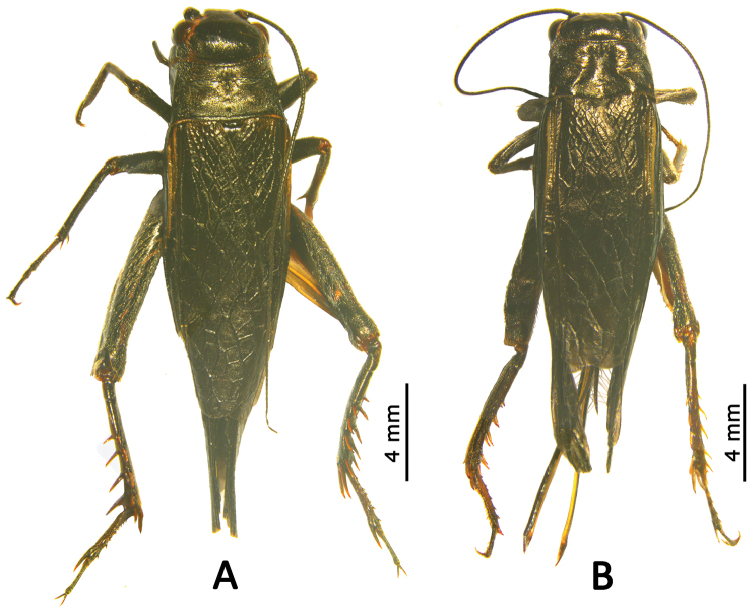
Habitus of *Qingryllus jiguanshanensis* sp. n., dorsal view. **A** male **B** female.

**Male**: Body medium, slightly small (Figure 2A). Head globular, smooth, with few pubescence. Frontal rostrum slightly obvious, dorsal surface flat, 1.8 times as wide as the scapus; eyes slightly protruding, rounded, located in latero-anterior sides of head; ocelli slightly rounded, median ocellus very small, lateral ocelli distinctly large; third joint of maxillary palpus distinctly long, 1.7 times as long as 4^th^ joint, slightly shorter than 5^th^ joint, 5^th^ joint widened apically, apical margin transversal and arc-shaped; last joint of labial palpus slightly longer than 2^th^ joint, slightly widened at apex, apical margin obtuse. Pronotum transverse, anterior margin slightly concave, nearly as wide as the head, distinctly widened posteriorly, posterior margin nearly straight, 1.3 times as wide as the anterior margin, 2.1 times wider than the length of pronotum; centre of pronotal disc with inconspicuous longitudinal furrow, the lateral sides with crescent impressions, lateral margins angularly rounded bent into paranota; lateral lobes distinctly smooth, without tomenta, anterior lower angles right angled, terminal obtuse, distinctly rising backwards, posterior lower angles wide, rounded. Tegmina slightly overlapping abdominal apex, with several irregular longitudinal veins and numerous transverse veins, forming irregular reticulum. Hind wings distinctly overlapping tegmina. Fore legs smooth, with no tympanum and no impressions on tibiae. Hind tibiae with four pairs of dorsal spines and three pairs of apical spurs on both sides, of which inner, upper and medial apical spurs are equal in length and distinctly longer than lower apical spurs; outer, upper and lower apical spurs slightly shorter than middle ones, nearly equal in length. Dorsal area of first tarsi with 5–8 outer and 4–5 inner spinules.

**Figure 3.**
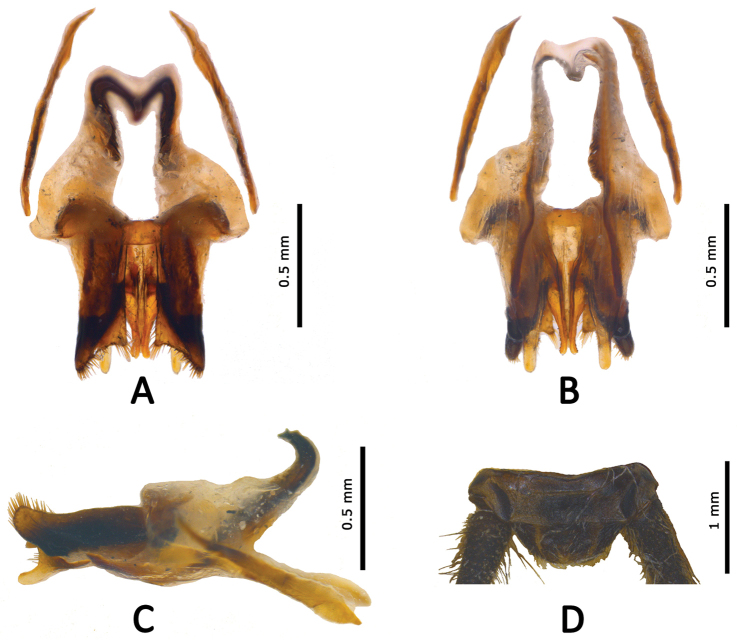
*Qingryllus jiguanshanensis* sp. n. Male. **A–C** genitalia (**A** dorsal view **B** ventral view **C** lateral view) **D** supra anal plate, dorsal view.

Supra anal plate (Figure 3D) semicircular, posterior margin broad, round. Subgenital plate rather long, narrowed posteriorly, coniform. Genitalia (Figure 3A–C). Epiphallus slightly longitudinal, anterior margin distinctly concave in middle; middle part of epiphallus narrowest, and distinctly widened anteriorly and posteriorly; apical part of epiphallus with two divided lobes, posterior margin slightly concave bearing setae. Ectoparamers semimembranous and long finger-like, not overlapping posterior margin of epiphallus; medial lobes short and slightly divided at apex. Endoparamers narrowed, reaching middle part of epiphallus. Apodeme distinctly developed and bend upward at apex.

**Figure 4.**
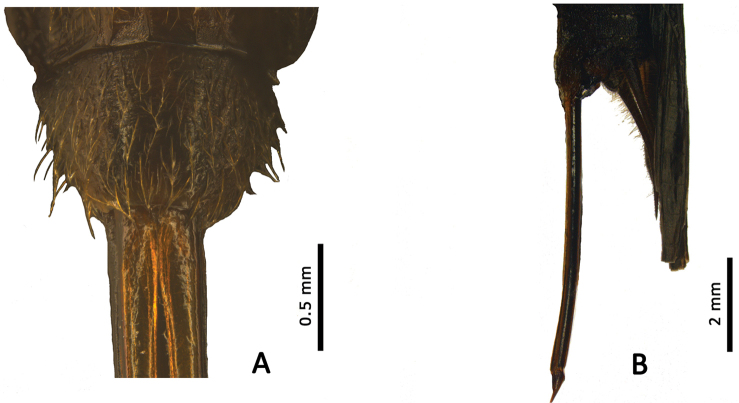
*Qingryllus jiguanshanensis* sp. n. Female. **A** Subgenital plate, ventral view **B** Ovipositor, lateral view.

**Female** (Figure 2B): Body very similar to that of male. Subgenital plate (Figure 4A) wide and short, narrowed posteriorly, posterior margin nearly straight. Ovipositor (Figure 4B) long and straight, near the end not swollen, apex acute.

Colouration. Body black brown to black. Part of 4^th^and 5^th^ joint of maxillary palpus, superior border of eyes, vein of tegmina on lateral sides and lower sides of hind femur yellowish-white.

#### Measurements

(**mm**). Male: body 11.2–12.2, length with wings 17.0–17.5, pronotum 1.9–2.0, tegmen 9.5–9.6, hind femur 7.0–7.5; Female: body 11.0, length with wings 16.0, pronotum 2.0, tegmen 9.5, hind femur 7.0, ovipositor 9.5.

#### Diagnosis.

This new species is similar to the type species, but differs from the latter by the posterior margin of pronotum being distinctly widened; veins of tegmina yellowish-white only on lateral side of dorsal area (between dorsal and lateral area of tegmina); epiphallus distinctly widened posteriorly. In the type species, the posterior margin of pronotum being slightly widened; basal part of dorsal area of tegmina also yellowish-white; epiphallus distinctly narrowed at apex.

#### Distribution.

China (Sichuan).

#### Etymology.

The specific name is derived from its type locality, Jiguanshan (China: Sichuan).

## Supplementary Material

XML Treatment for Qingryllus jiguanshanensis
